# A facile ultrasonic-assisted fabrication of nitrogen-doped carbon dots/BiOBr up-conversion nanocomposites for visible light photocatalytic enhancements

**DOI:** 10.1038/srep45086

**Published:** 2017-03-22

**Authors:** Yifan Zhang, Mira Park, Hak Yong Kim, Bin Ding, Soo-Jin Park

**Affiliations:** 1 Department of Chemistry and Chemical Engineering, Inha University, 100 Inharo, Incheon 402-751, South Korea; 2 Department of Organic Materials and Fiber Engineering, Chonbuk National University, Jeonju 561-756, South Korea; 3 Department of BIN Convergence Technology, Chonbuk National University, Jeonju 561-756, South Korea; 4 State Key Laboratory for Modification of Chemical Fibers and Polymer Materials, College of Textiles, Donghua University, Shanghai 200051, China

## Abstract

A highly efficient novel photocatalyst consisting of nitrogen-carbon dots (N-CDs) and three-dimensional (3D) hierarchical BiOBr was synthesized via a simple ultrasonic-assisted method and used for the degradation of hazardous dyes. Deposition of N-CDs onto the surface of BiOBr was confirmed through structure and composition characterizations. The N-CDs/BiOBr composites exhibited superior activity for organic contaminant degradation under visible light and the 1 wt% N-CDs/BiOBr composite showed the highest degradation rate, indicating that N-CDs/BiOBr composites have great potential for application in mitigating hazardous contaminants. The N-CDs played an important role in improving the photocatalytic performance, owing to the enhancement of up-converted photoluminescence behavior as well as the efficient separation of photogenerated charge carriers originating from the intimately contacted interface. A possible photocatalytic mechanism was proposed based on the experimental results.

Carbon-based quantum dots consisting of graphene quantum dots (GQDs) and carbon quantum dots (CQDs, C-dots or CDs) are a new class of carbon nanomaterials with sizes below 10 nm^1^. Carbon dots (CDs), a commonly known new type of carbon nanoparticles, have drawn much attention owing to their advantages over conventional semiconductor-based quantum dots, such as their low-cost, high chemical stability, excellent water solubility, low toxicity and good biocompatibility^2^,^3^,^4^,^5^,^6^,^7^,^8^. Owing to their unique structural, optical and biochemical features, these materials have been studied extensively for application in chemical sensing, photocatalysis, bioimaging, fluorescent markers and optical sensors^9^,^10^,^11^,^12^,^13^,^14^. Considering these attractive and unique advantages, CDs are increasing in popularity as new-generation carbon nanomaterials.

There are two approaches for synthesizing CDs, classified as “top-down” and “bottom-up”^15^,^16^,^17^,^18^. The first route involves carving larger graphitic materials into smaller carbon nanoparticles via physical or chemical approaches. Compared to the “top-down” method, the “bottom-up” approach is more attractive for harvesting CDs from a variety of molecular precursors through chemical synthesis or carbonization. Methods include chemical or thermal oxidation of suitable chemical precursors, ultrasonic treatment of alkaline oracidic aqueous solutions of glucose and microwave pyrolysis of carbohydrates^19^,^20^,^21^,^22^,^23^.

Nitrogen-doped carbon dots (N-CDs), a novel carbon material doped with nitrogen atoms, have attracted significant research interest owing to their photochemical properties, electrocatalytic activity, biocompatibility and up-conversion properties. Because of these properties, N-CDs have been applied in many fields such as electrochemistry, photocatalysis^24^,^25^, and optical devices. For instance, Teng *et al*.^26^ reported that N-CDs work as photocatalysts for hydrogen production under visible light, and Chen *et al*.^27^ reported that N-CDs can be used as probes for the detection of Hg^2+^ ions.

Recently, the semiconductor BiOBr has attracted considerable research interest in photocatalysis owing to its excellent electrical and optical properties^28^. However, the band gap of BiOBr is approximately 2.9 eV, so it does not absorb a significant portion of visible light. In order to enhance its visible-light absorption, many attempts have been made to combine it with other materials such as another semiconductor or a carbon-based material.

Many strategies have been applied for the preparation of N-CD/semiconductor composites. However, all the methods reported so far suffer from drawbacks such as high cost, and the need for high pressure and complicated, time-consuming procedures^29^,^30^.

In this study, we have developed a new method to combine N-CDs with BiOBr (BCs). To our knowledge, such a method has not been reported previously. The N-CDs/BiOBr composites were synthesized by a facile ultrasonic-assisted method. The experimental results indicated that the as-prepared composites have higher photocatalytic activity and stability than pure BiOBr.

## Experimental

### Chemicals

Bi(NO_3_)_3_·5H_2_O, KBr, Rhodamine B(RhB), ethylenediamine (EDA), citric acid (CA) Tert-Butanol (TBA), ethylenediaminetetraacetic acid disodium salt (EDTA-2Na), benzoquinone (BQ) and AgNO_3_ were purchased from Sigma Aldrich. Ultra-pure water was prepared by using a water purification system. All the initial chemicals in this work were used without further purification.

### Sample preparation

In a typical synthesis, 0.02 mol of KBr was dissolved in 150 mL of H_2_O. To the above solution, 0.02 mol of Bi(NO_3_)_3_·5H_2_O was added slowly. The pH was adjusted to 10 using 1 M NH_3_·H_2_O, and the mixture was stirred for 12 h. The resulting solid was then collected, washed several times with water, and dried at 60 °C to afford the final sample.

To prepare the N-CDs, 2.1 g (10 mmol) CA and 1.8 g (30 mmol) EDA were dissolved in 50 mL water, and the mixture was stirred to form a clear solution. The solution was then transferred to a 750 W microwave oven. The solution was heated and kept for an additional 9 min. The final product was collected by adding deionized water and centrifuging at 5000 rpm for 30 min to remove the large particles. To further purify the N-CDs, they were added to water and subjected to dialysis against pure water using a 1 kDa molecular weight cut-off membrane for 24 h. A clear yellow-brown solution was obtained. Finally, the product in the dialysis membrane was obtained using a freeze-dryer. The dried product was stored in a refrigerator prior to use.

A simple and mild method was used to synthesize the N-CD/BiOBr samples. First, predetermined amounts of N-CDs and BiOBr were dissolved in ultrapure water. Then the solution was ultrasonicated using a strong bar-type ultrasonic device (Fig. S1) for 30 min. After washing with deionized water and ethanol five times to remove impurities. The as prepared samples were stored in an oven for 6 h. N-CD/BiOBr composites with different mass ratios of N-CDs to BiOBr (0.005, 0.01, 0.015, 0.02, 0.03, and 0.04) were prepared according to the above procedure, and the products were labeled as BC-0.5, BC-1, BC-1.5, BC-2, BC-3, and BC-4, respectively. Pristine BiOBr nanoplates were also prepared using the same method without the addition of N-CDs and were named BC-0. The as-prepared samples are shown in Fig. S2.

### Photocatalytic activity measurement

Photocatalytic activities of the as-prepared samples were estimated by the degradation rate of RhB under a Solar Simulator (Sun 2000, ABET) with a 440 nm cutoff filter. All photocatalytic experiments were carried out at room temperature. In a typical photocatalytic experiment, 0.01 g of photocatalyst was dispersed into 50 mL of the RhB (10 ppm) solution. Before irradiation, the solution was continuously stirred for 1 h in the dark to make sure that the absorption-desorption equilibrium had been achieved, and then irradiation procedures were conducted. After that, 4 mL solution was withdrawn and analyzed with a UV-Vis spectrophotometer at 553 nm to measure its absorbance.

For the radical scavengers experiments, 0.01 g of photocatalyst was dispersed into 50 mL of the RhB (10 ppm) solution, and then 20 mg^31^,^32^ of TBA, EDTA, BQ and AgNO_3_ were used as h^+^(holes) scavenger, •OH scavenger, 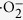 scavenger and e^−^ scavenger, respectively. Before irradiation, the solution was continuously stirred for 1 h in the dark to make sure that the absorption-desorption equilibrium had been achieved, and then irradiation procedures were conducted. After that, 4 mL solution was withdrawn and analyzed with a UV-Vis spectrophotometer at 553 nm to measure its absorbance.

### Up-conversion PL emission measurement

Up-conversion PL properties of the as-prepared N-CDs were estimated by a LS55 Fluorescence Spectrometer (PerkinElmer, USA). All experiments were carried out at room temperature. In a typical up-conversion PL experiment, 0.5 mg/ml of N-CDs solution was prepared by dissolving 5 mg of N-CDs in 10 ml of distilled water and sonicated for 1 h. And then, 0.5 mg/ml of N-CDs solution was moved to fluorescence fpectrometer to analyze. Excitation wavelength were set to 675, 700, 725, 750, 775 and 800 nm, respectively.

### Characterization

The structures of the samples were investigated using X-ray diffraction (XRD, D2 PHASER, Bruker). Atomic force microscopy (AFM) images were obtained using a Bruker Nanoscope with a Multimode IVa controller. The N-CD size distribution and potential were investigated using a zeta potential analyzer (Nanotrac wave, Microtrac). The morphology was evaluated using scanning electron microscopy (SEM, Model SU8010, Hitachi Co., Ltd.) and field-emission transmission electron microscopy (FE-TEM) images were acquired using a JEM-2100F instrument. X-ray photoelectron spectroscopy (XPS, VG Scientific Co., ESCA LAB MK-II) was used. An adsorption analyzer (BEL BELSORP) was used to obtain the specific surface areas of the samples. Infrared spectra were acquired with a Fourier-transform infrared (FT-IR) spectrometer (FT-IR 4200, JASCO, USA). Photoluminescence (PL) emission spectra were acquired under excitation at 325 nm. The excitation and emission spectra of the N-CDs are recorded by a LS55 Fluorescence Spectrometer (PerkinElmer, USA). Electrochemical impedance spectroscopy (EIS) was carried out using an Ivium electrochemical analyzer with a conventional three-electrode system where modified nickel foam, a Ag/AgCl electrode, and a platinum wire were used as the working electrode, reference electrode, and counter electrode, respectively. A 3 M KOH solution was used as the electrolyte, and a frequency range of 0.1 Hz to 10 kHz was employed. UV-Vis absorbance spectroscopy was performed using a spectrometer fitted with an SA-13.1 diffuse reflector (S-3100, Scinco Co., Ltd.).

## Results and Discussion

### Crystal structures of samples

**Figure 1:**
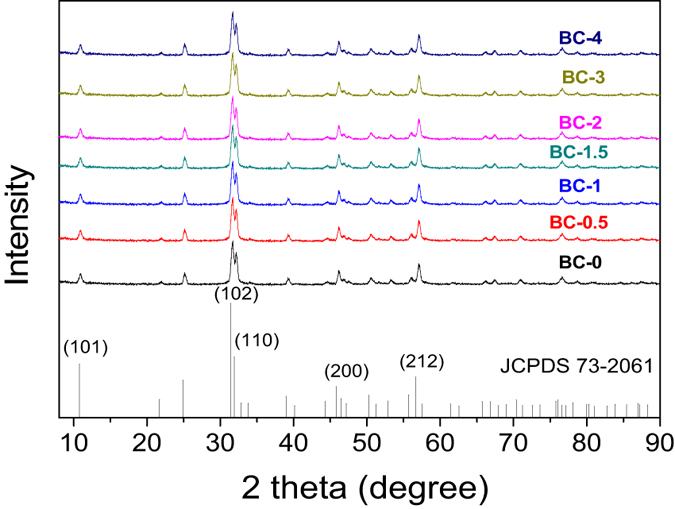
XRD patterns of as prepared samples.

XRD is an effective method for evaluating crystalline properties. As shown in Fig. 1, BiOBr (JCPDS No. 73-2061) is a well-known typically crystalline material that exhibits strong reflections in its XRD spectra. All the diffraction peaks can be indexed to BiOBr with well-resolved (101), (102), (110), (200), and (212) planes^33^,^34^. However, no diffraction peaks corresponding to N-CDs are observed in the BC samples, because the small amount of N-CDs present cannot be resolved by XRD. No other diffraction peaks are observed, indicating that the as-obtained samples are very pure and that no impurities are formed.

### FT-IR analysis

**Figure 2:**
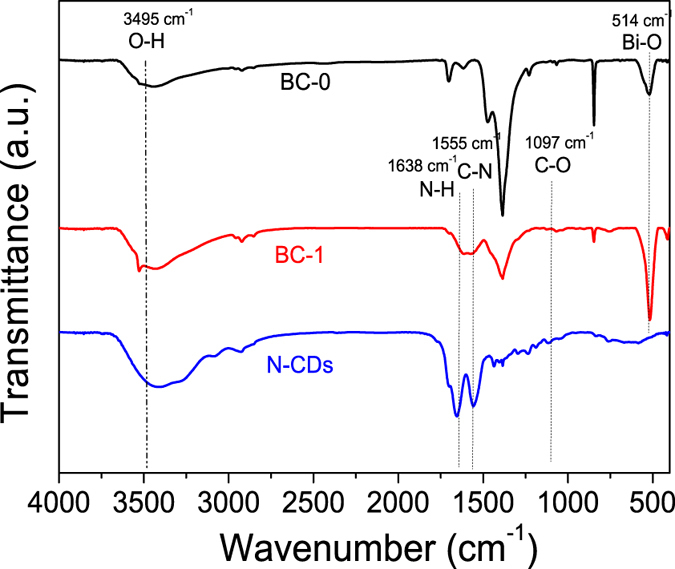
FT-IR spectra of samples: (**a**) BC-0, (**b**) BC-1, (**c**) N-CDs.

FT-IR analysis was conducted to further confirm the formation of the BC samples. The FT-IR spectra of BC-0, BC-1, and the N-CDs are shown in Fig. 2. The peak located at 3495 cm^−1^ is related to O-H stretching, and is ascribed to adsorbed water. The FT-IR spectra of BC-0 and BC-1 exhibit peaks at 514 cm^−1^, which are related to the Bi-O stretching vibration. In the spectrum of BC-1, stretching vibrations for N-H at 1638 cm^−1^ and C-N at 1400 cm^−1^ can be clearly observed, which may indicate successful adulteration with N-CDs. Furthermore, the absorption band at 1078 cm^−1^ is attributed to the stretching vibration of C-O^35^,^36^. After BiOBr is combined with the N-CDs, all the above absorption bands are be observed in the BC-1 nanohybrid, which indicates that BiOBr and N-CDs are coupled successfully. All of the XRD and FT-IR results indicate that BC-1 contains BiOBr and N-CDs as the two fundamental components, and that a chemical reaction occurred between BiOBr and N-CDs.

### XPS analysis

**Figure 3:**
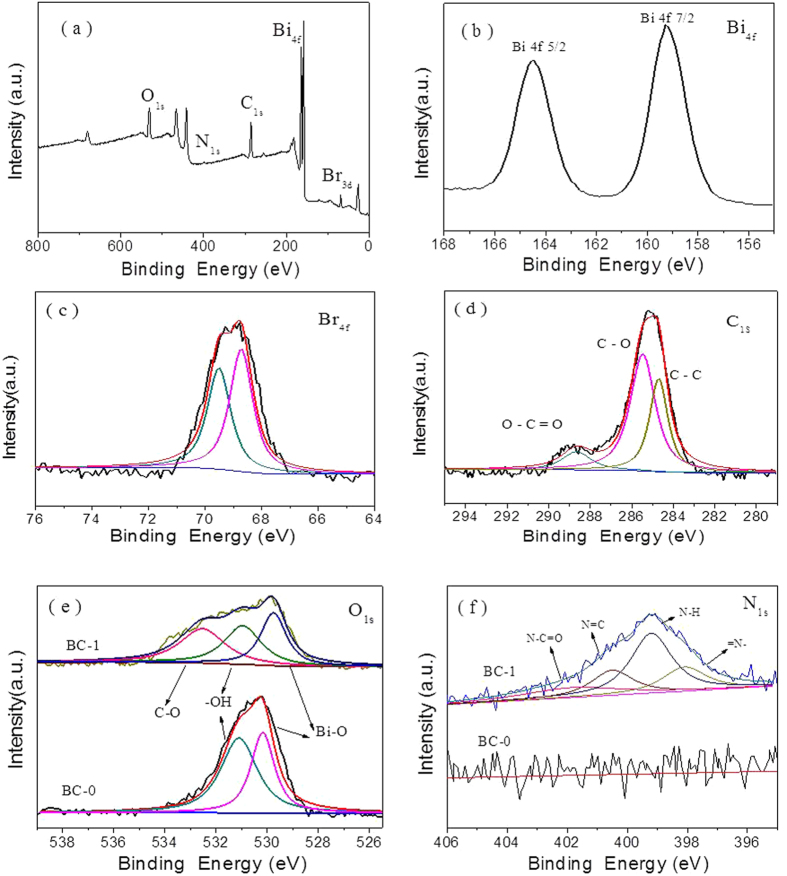
XPS spectra of the as-prepared samples: (**a**) survey of BC-1, (**b**) Bi_4f_ of BC-1, (**c**) Br_4f_ of BC-1, (**d**) C_1s_ of BC-1, (**e**) O_1s_ of BC-0 and BC-1, and (**f**) N_1s_ of BC-0 and BC-1.

More information on the chemical environment of BiOBr and the N-CDs was obtained through XPS. Figure 3(a) shows the full survey spectrum of BC-1. Figure 3(b) shows peaks with binding energies of 159.7 and 165.0 eV, corresponding to Bi_4f7/2_ and Bi_4f5/2_, respectively. Figure 3(c) shows peaks with binding energies of approximately 68.7 and 69.5 eV, which can be ascribed to Br_4f_. The XPS spectra for the C_1s_ region at approximately 285 eV are shown in Fig. 3(d). The peak at a binding energy of 284.7 eV is attributed to C–C bonds and is identified as originating from an amorphous carbon phase or from adventitious carbon. The peak of the carboxyl carbon (O–C=O) is located at 288.67 eV. Moreover, the peak at 285.5 eV is characteristic of C–O groups^37^,^38^. Figure 3(e) shows the O_1s_ spectra of BC-0 and BC-1. The wide and asymmetric peak in the O_1s_ spectrum indicates that there may be more than one chemical state with a similar binding energy. The peaks at 529.79, 531.0, and 532.6 eV are related to Bi–O, bridging hydroxyls, and oxygen singly bonded to carbon (C–O), respectively. Upon comparison with BC-0, one more peak located at 532.6 eV is observed. This is evidence of bonding between BiOBr and the N-CDs. N1s XPS-peak-differentiation-imitating analyses of the spectrum in Fig. 3(f) indicates that there are four chemical forms of N: = N– (397.4 eV), N–H (399.0 eV), N = C (399.0 eV), and N–C = O (399.0 eV). Taken together, these results indicate that the heterostructures are composed of BiOBr and N-CDs.

### Physical structures of samples

**Figure 4:**
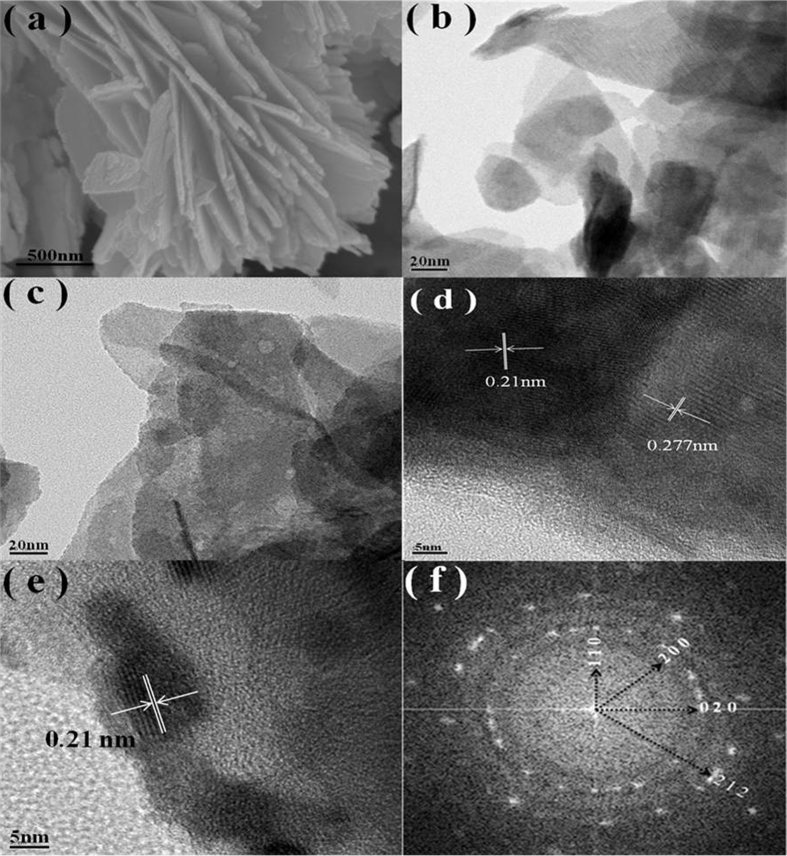
(**a**) SEM image of BC-1. (**b**) FE-TEM image of BC-0 and (**c**) BC-1. (**d**,**e**) FE-TEM image of BC-1 nanohybrids. (**f**) Fast Fourier transform (FFT) from the image of (**d**).

**Figure 5:**
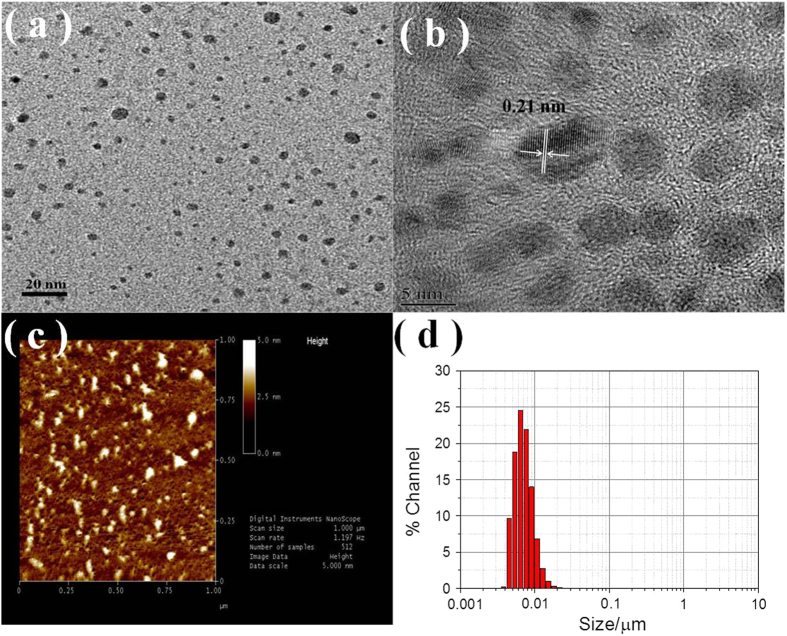
(**a**,**b**) FE-TEM image of N-CDs. (**c**) AFM images of the N-CDs and their height distribution. (**d**) Accurate N-CDs particle size distribution measured by Zeta Potential device.

The morphologies of the samples were observed using SEM. Figure 4(a) shows the SEM images of BC-1. It can be seen that the BiOBr nanoplates are irregular and overlap with each other. The FE-TEM images of the BC-0 and BC-1 nanohybrids are shown in Fig. 4(b and c). Compared to BC-0, the BC-1 nanohybrids are rougher. Therefore, it can be deduced that BiOBr and N-CDs are in close contact with each other. Furthermore, the FE-TEM images of the BC-1 nanohybrid presented in Fig. 4(d and e) show lattice fringes of a highly crystalline species. The lattice spacing of the nanoplates of around 0.277 nm is ascribed to the (110) lattice plane of BiOBr, and N-CDs with a lattice spacing of 0.21 nm are observed. In addition, the XRD patterns of the N-CDs are shown in Fig. S3, and correspond with the FE-TEM image. Thus, it is reasonable to conclude that the BiOBr/N-CD nanohybrids were successfully synthesized via the facile ultrasonication-assisted method. Furthermore, in Fig. 4(e), we can observe that the BiOBr nanoplates are decorated with N-CDs with an average diameter of 5–7 nm, which corresponds with Fig. 5(b,c and d). Moreover, the corresponding fast Fourier transform (FFT) pattern in Fig. 4(f) shows a regular spot pattern, confirming the single-crystalline nature of BiOBr. The angle labeled in the FFT pattern is 45°, which is in agreement with the theoretical value for the angle between the (110) and (200) planes. Additionally, (020) and (212) planes can also be detected, indicating four different crystal layers with different rotational angles.

### Analysis of N-CDs particle size and potenial

As shown in Fig. 5(a), the FE-TEM images indicate that the N-CDs are well dispersed and have an average diameter of 5 nm. Figure 5(b) reveals that the lattice spacing of the N-CDs is approximately 0.21 nm, which agrees well with the (002) spacing of graphitic carbon and is similar to those of other previously reported N-CDs^39^. Figure 5(c) shows that the N-CDs are scattered well on the silicon wafer, and the topographic heights are mainly approximately 5 nm. The N-CD diameter statistics obtained using an accurate particle size zeta potential analyzer are shown in Fig. 5(d). Once again, the results confirm a normal distribution curve centered around 5 nm. The obtained zeta potential values of the N-CDs, BC-0, and BC-1 are given in Fig. S4. As expected, the potentials of the N-CDs, BC-0, and BC-1 are 0.5, −1.6, and −0.8 mV, respectively. This is evidence for the mechanism of the combination of the N-CDs and BiOBr. Owing to the positive and negative charge properties revealed by the zeta potentials, the N-CDs and BiOBr can be combined easily.

### Specific surface area analysis

**Figure 6:**
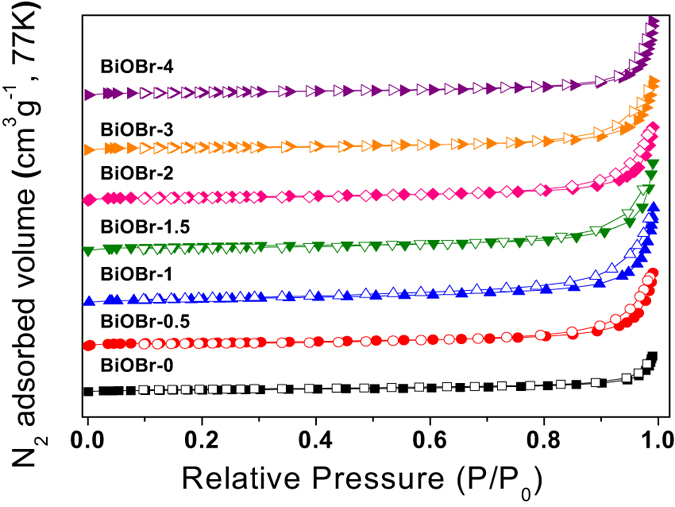
N_2_ adsorption/desorption isotherms of as-prepared samples.

The Brunauer-Emmett-Teller (BET) surface areas of the as-prepared samples were investigated using the nitrogen adsorption/desorption method. As shown in Fig. 6 and Table 1, typical isotherms with hysteresis loops are obtained. The largest hysteresis loop is observed for BC-1, which is in agreement with its highest specific surface area (37.484 m^2^ g^−1^). The specific surface area of BC-0 is only 15.323 m^2^ g^−1^. Thus, we can deduce that the N-CDs effectively provide a larger surface area. As shown in Fig. S5, we measured the surface area of N-CDs, which was 7.215 m^2^ g^−1^. The specific surface area of BC-1 is higher than those of as-prepared BC-0.5, BC-1.5, BC-2, BC-3, and BC-4. These results suggest that an appropriate amount of N-CDs provides more active sites for photocatalytic reactions. As shown in Table 1, the pore volumes of the composite samples increase from 0.1104 to 0.2711 cm^3^ g^−1^. BC-1 has a pore volume of 0.2711 cm^3^ g^−1^, which is approximately 2.5 times larger than that of BC-0. As we know, a high surface area and pore volume leads to more surface active sites and effective reactant transport, providing an enhancement of photocatalytic performance. Thus, these results suggest that BC-1 could provide enhanced catalytic performance.

### Investigation of enhanced photocatalytic performance

**Figure 7:**
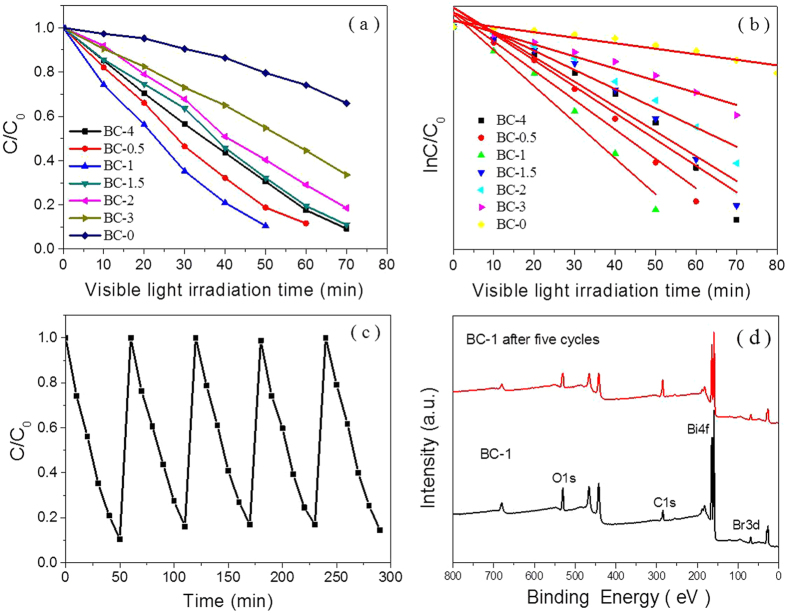
(**a**) Photodegradation performance of as-prepared samples and (**b**) reaction rate correlation of the degradation reaction of as-prepared samples. (**c**) Cycling curve for photocatalytic degradation of BC-1. (**d**) XPS patterns of BC-1 and BC-1 after five cycles.

As prepared samples were used as photocatalytic references in the experiment. Figure 7(a,b) and Table 2 show the degradation curves and rate constants. Within 50 min of reaction, the photodegradation efficiencies of BC-0, BC-0.5, BC-1, BC-1.5, BC-2, BC-3 and BC-4 reach approximately 70.0%, 80.9%, 89.3%, 67.1%, 59.6%, 45.0% and 20.0%, respectively. Within 50 min, 89.3% of the RhB is removed by BC-1 under visible light irradiation, which is almost 19.3% higher than that of BC-0 and 4.4 times higher than that for BC-4. The enhanced photocatalytic performance of the BC-1 composite may be due to the following correlated reasons: (1) The larger BET surface area and pore volume facilitates more efficient contact of the hierarchical N-CDs with BiOBr; (2) the unique hierarchical heterostructures allow for more efficient use of the light source, leading to greatly enhanced visible light photocatalytic properties; (3) the adopted fabrication route successfully provides close contact between BiOBr and the N-CDs. To examine its stability, BC-1 was used in repeated photodegradation cycles of RhB. As shown in Fig. 7(c,d) and Fig. S6, each experiment was carried out under identical conditions. After five cycles, the photocatalytic activity, crystal structure and chemical environment of BC-1 remain almost unchanged, clearly indicating its stability.

**Figure 8:**
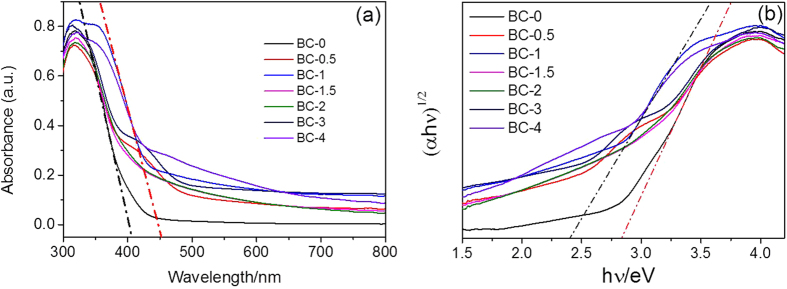
(**a**) Diffuse reflectance spectra of as prepared samples. (**b**) The bandgap value, estimated by a related curve of (αhν)^1/2^ versus photon energy plotted.

The optical absorbances of the samples were measured using diffuse reflectance spectra (DRS). Figure 8(a) shows the DRS spectra of the as-prepared products. The absorbance edges of BC-0 and BC-1 are located at 400 and 442 nm, respectively. Meanwhile, the bandgap value of as prepared samples shifted from 2.85 to 2.41 eV (Fig. 8(b)). Combined with the results of optical measurements, we can deduce that BC-1 has better visible light absorption properties.

**Figure 9:**
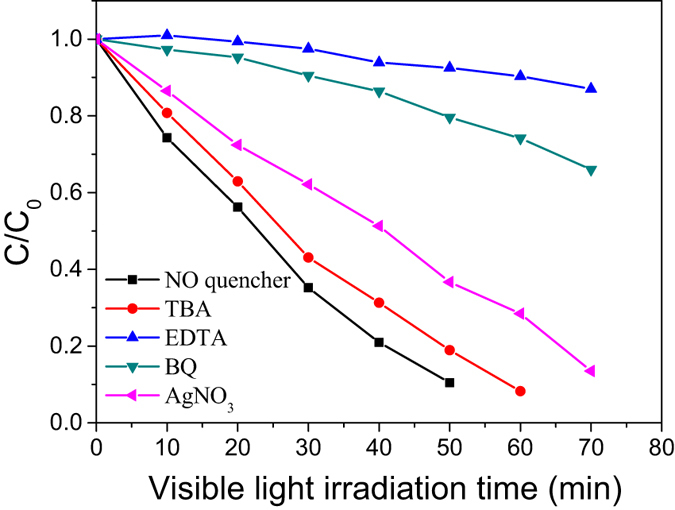
Degradation of RhB over BC-1 in the presence of scavengers.

To explore the photocatalytic mechanism, scavenging analysis was performed using different scavengers to remove specific reactive species. EDTA-2Na was used as a scavenger for h^+^(holes), AgNO_3_ for e^−^, BQ for 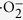 and TBA for •OH. Figure 9 shows the photocatalytic degradation of RhB over BC-1 in the presence of different scavengers under visible-light irradiation. The photodegradation efficiency of RhB decreases only slightly with the addition of TBA and AgNO_3_, indicating that •OH and e^−^ are not the dominant active species in this process. However, with the addition of EDTA-2Na and BQ, the photodegradation of RhB decreases dramatically, suggesting that h^+^ and 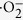 play key roles in the photocatalytic process.

**Figure 10:**
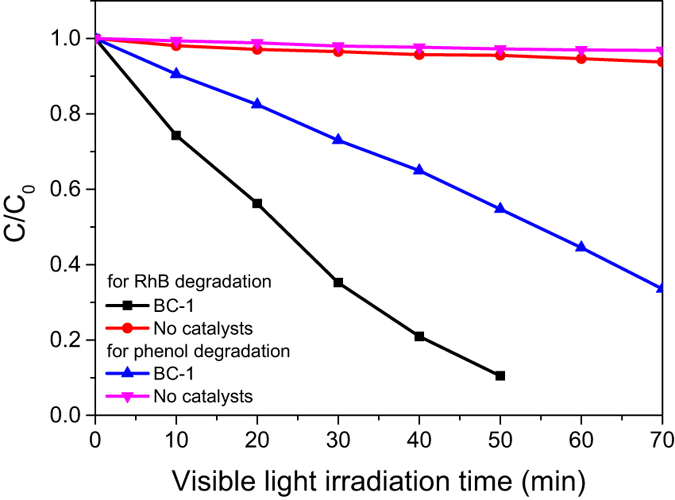
Comparison of photocatalytic activity of BC-1 on phenol and RhB under visible light irradiation.

In order to confirm the dye photosensitization on the enhanced visible-light-driven photodegradation of RhB, we conducted a parallel experiment of photodegradation of phenol under visible light irradiation. Results are shown in Fig. 10. In the absence of photocatalysts, degradation of phenol and RhB under visible light irradiation is inappreciable, suggesting that the photodegradation of phenol and RhB is negligible. In the presence of BC-1, when compared the degradation performance with each other, the degradation of phenol becomes distinct. Because the phenol can’t absorb visible light, so the dye photosensitization degradation process can’t take place. It is clear that the degradation rate of RhB is much more quick than that of phenol, so we can obtain that the dye photosensitization plays a crucial role in the degradation of dyes under visible light. Based on the scavengers and dye photosensitization experimental results, the degradation procedure can be described as follows:


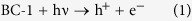



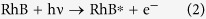



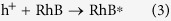



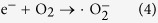



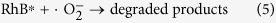


**Figure 11:**
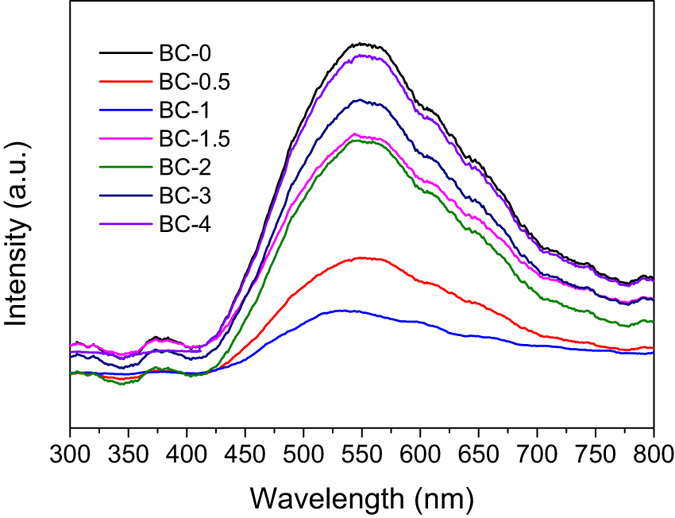
PL emission spectra for as prepared samples.

PL emission spectra obtained from the recombination of photoinduced charge carriers are a useful tool to explore the enhancement of charge transportation and separation. The PL signals for as-prepared are given in Fig. 11, lower PL intensity of BC-1 indicates a lower recombination rate for the photoinduced electron-hole pairs, which is consistent with the photocatalytic activity of the samples. For BC-1, the recombination of the electron-hole pairs is effectively inhibited, leaving more charge carriers to form reactive species and promote the degradation of the pollutants. During the reaction, the photoinduced holes migrate to the nearby RhB and take part in the oxidative photodegradation. Simultaneously, electrons are easily transferred to the N-CDs and then captured by O_2_ for further reactions. Hence, a higher reaction rate is achieved.

**Figure 12:**
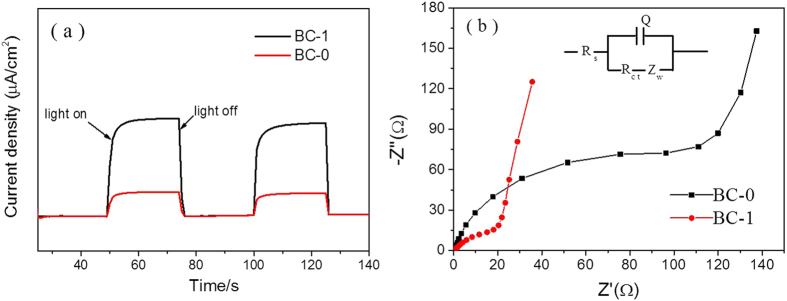
(**a**) Transient photocurrent response for BC-0 and BC-1, (**b**) EIS spectra of BC-0/Nickle foam and BC-1/Nickle foam.

The transient photocurrent method is regarded as an efficient method to explain the e^−^/h^+^ recombination rate. As shown in Fig. 12(a), irradiation with visible light caused a rapid increase of the intensity of the photocurrent, the photocurrent returned to zero when irradiation was discontinued. BC-0 exhibited a lower photocurrent density. Moreover, BC-1 exhibited a much higher photocurrent response, which is indicative of higher efficiency of separation of the e^−^ and h^+^. This is beneficial to the photocatalytic reaction, and the higher photocurrent response indicates that CDs can efficiently enhance the migration efficiency of photoinduced electrons, which was in agreement with the PL emission spectra (Fig. 11). Meanwhile, as shown in Fig. 11, with the increasing of CDs percentage, the PL intensity became higher than BC-1. We can deduce that there is a shielding effect, when the CDs were aggregated with each other.

On the other hand, to further investigate the charge transfer resistance of as-prepared samples, BC-0 and BC-1 were investigated using EIS. From Fig. 12(b), it could be deduced that the charge-transfer resistance (R_ct_) values of BC-0 and BC-1 were 20.2 and 111.2 Ω, respectively. We could conclude that the combination of N-CDs with BiOBr improve the electron transport ability of BiOBr. On the other hand, the lower R_ct_ of BC-1 indicated that enhanced separation of photo-generated electrone-hole pairs and faster interfacial charge transfer had been achieved.

**Figure 13:**
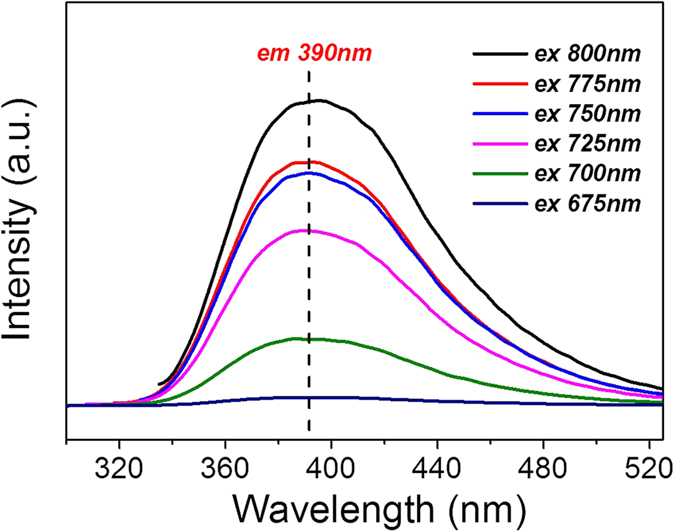
Up-converted PL spectra of the N-CDs at with excitation of different visible-near infrared wavelengths.

**Figure 14:**
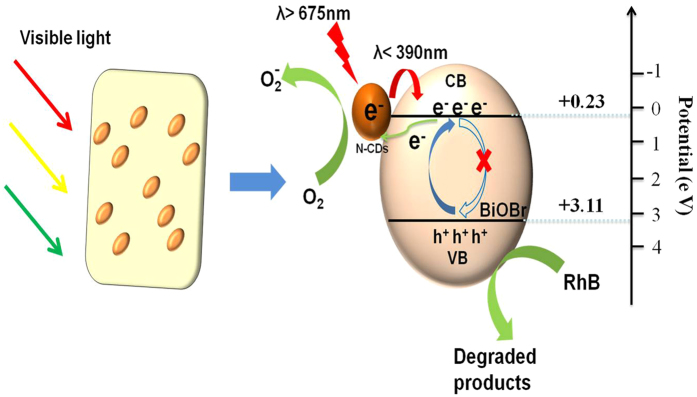
Schematic diagram representing the photocatalytic reaction.

In order to explore the PL properties of the as-prepared N-CDs, a systematic study was performed using near-infrared (NIR) excitation. As shown in Fig. 13, when the N-CDs are excited by long-wavelength light (from 800 to 675 nm), the up-converted emission wavelengths are located at around 390 nm. It proves that up-conversion PL properties of as prepared N-CDs, which will result in short wavelength emission at 390 nm. Based on these results, we conclude that the N-CDs also show an excitation-independent PL behavior. Furthermore, using the results of this experiment, a hierarchical mechanism for the BC composite samples was constructed. Figure 14 shows the mechanism proposed to explain the enhancement of the photocatalytic properties of the BCs. As is well known, BiOBr exhibits better absorption of light with wavelengths below 400 nm. After the N-CDs are combined with the BiOBr, a larger percentage of visible light is converted to shorter wavelengths. As a result, photocatalytic enhancement is achieved.

## Conclusions

In summary, novel BiOBr/N-CD nanocomposites were prepared by a facile ultrasonic-assisted method based on contact between BiOBr and N-CDs. The as-prepared samples were demonstrated to have nanosized interfacial contacts and the BiOBr/N-CD nanohybrids proved to have enhanced visible-light photoactivity and photostability. The loading amount of N-CDs had a significant influence on the photoactivity of BiOBr; the optimal ratio of N-CDs to BiOBr was 0.01. The mechanism of the catalyst enhancement was discussed in detail. The improved photocatalytic activity was mainly attributed to the superior electron-transfer ability of the N-CDs and their upconverted photoluminescence behavior, which enhanced light harvesting. The main active species were determined to be holes and superoxide radicals under visible-light irradiation through free-radical-trapping experiments. We hope that this research will attract more attention to N-CDs and semiconductor catalysts for application in photocatalytic water purification.

## Additional Information

**How to cite this article**: Zhang, Y. *et al*. A facile ultrasonic-assisted fabrication of nitrogen-doped carbon dots/BiOBr up-conversion nanocomposites for visible light photocatalytic enhancements. *Sci. Rep.* **7**, 45086; doi: 10.1038/srep45086 (2017).

**Publisher's note:** Springer Nature remains neutral with regard to jurisdictional claims in published maps and institutional affiliations.

## Supplementary Material

Supplementary Information

## Figures and Tables

**Table 1:** S_BET_ and pore volume of the as-prepared samples.

Specimens	aS_BET_ (m^2^/g)	bTotal pore volume (cm^3^/g)	cMicropore volume (cm^3^/g)
BC-0	15.3	0.11	0.04
BC-0.5	23.4	0.18	0.07
BC-1	37.5	0.27	0.13
BC-1.5	35.8	0.26	0.10
BC-2	28.5	0.22	0.09
BC-3	25.6	0.21	0.08
BC-4	24.7	0.23	0.07

^a^S_BET_: Specific surface area computed using BET equation.

^b^Total volume are obtained by BET equation.

^c^Micropore volume was obtained using the D-R equation.

**Table 2:** Rate constants and R^2^ of the as-prepared samples.

Specimens	Rate constants (min^−1^)
BC-0	0.0325
BC-0.5	0.0362
BC-1	0.0444
BC-1.5	0.0306
BC-2	0.0237
BC-3	0.0149
BC-4	0.0068
