# Supplementary material for: A facile ultrasonic-assisted fabrication of nitrogen-doped carbon dots/BiOBr up-conversion nanocomposites for visible light photocatalytic enhancements
Source: Sci Rep. 2017 Mar 22;7:45086. doi: 10.1038/srep45086 (PMC5361154; doi:10.1038/srep45086)
Supplement: Supplementary Information [file srep45086-s1.doc]

**Electronic Supplementary Information**

A facile ultrasonic-assisted fabrication of nitrogen-doped carbon dots/BiOBr up-conversion nanocomposites for visible light photocatalytic enhancements

Yifan Zhang,^a^ Mira Park,^b^ Hak-Yong Kim,^c^ Bin Ding,^d^ and Soo-Jin Park^a*^

*^a^ Department of Chemistry and Chemical Engineering, Inha University, 100 Inharo, Incheon 402-751, South Korea*

*^b^ Department of Organic Materials and Fiber Engineering, Chonbuk National*

*University, Jeonju 561-756, South Korea.*

*^c^ Department of BIN Convergence Technology, Chonbuk National University, Jeonju 561-756, South Korea.*

*^d^ State Key Laboratory for Modification of Chemical Fibers and Polymer Materials, College of Textiles, Donghua University, Shanghai 200051, China*

*Corresponding author. [Tel: +82-32-860-7234, Fax: +82-32-860-5604](tel:+82-32-860-7234; Fax:+82-32-860-5604)

E-mail address: [sjpark@inha.ac.kr (S. J. Park)](mailto:sjpark@inha.ac.kr (S. J. Park))


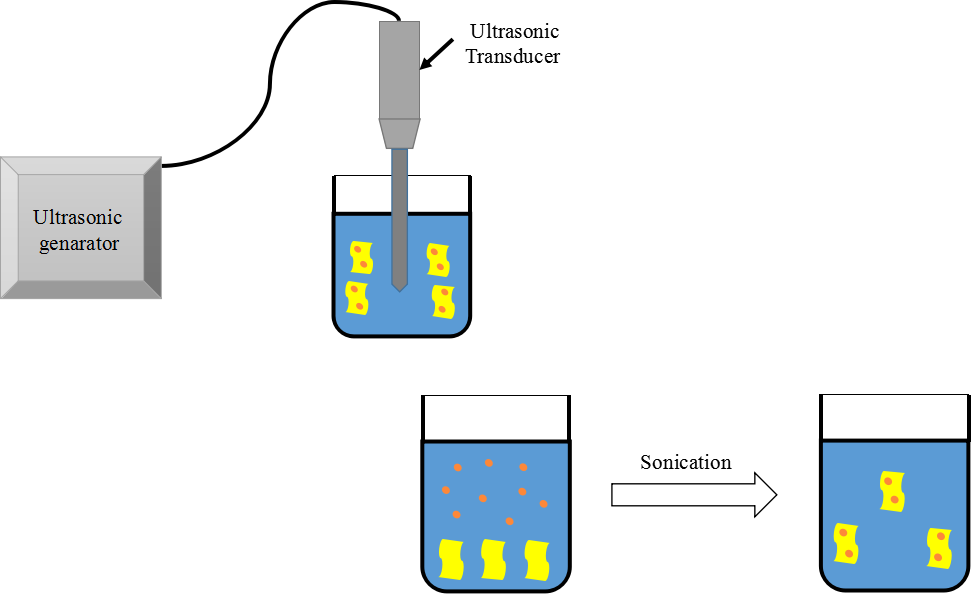


Figure. S1. Strong bar-type ultrasonic device.


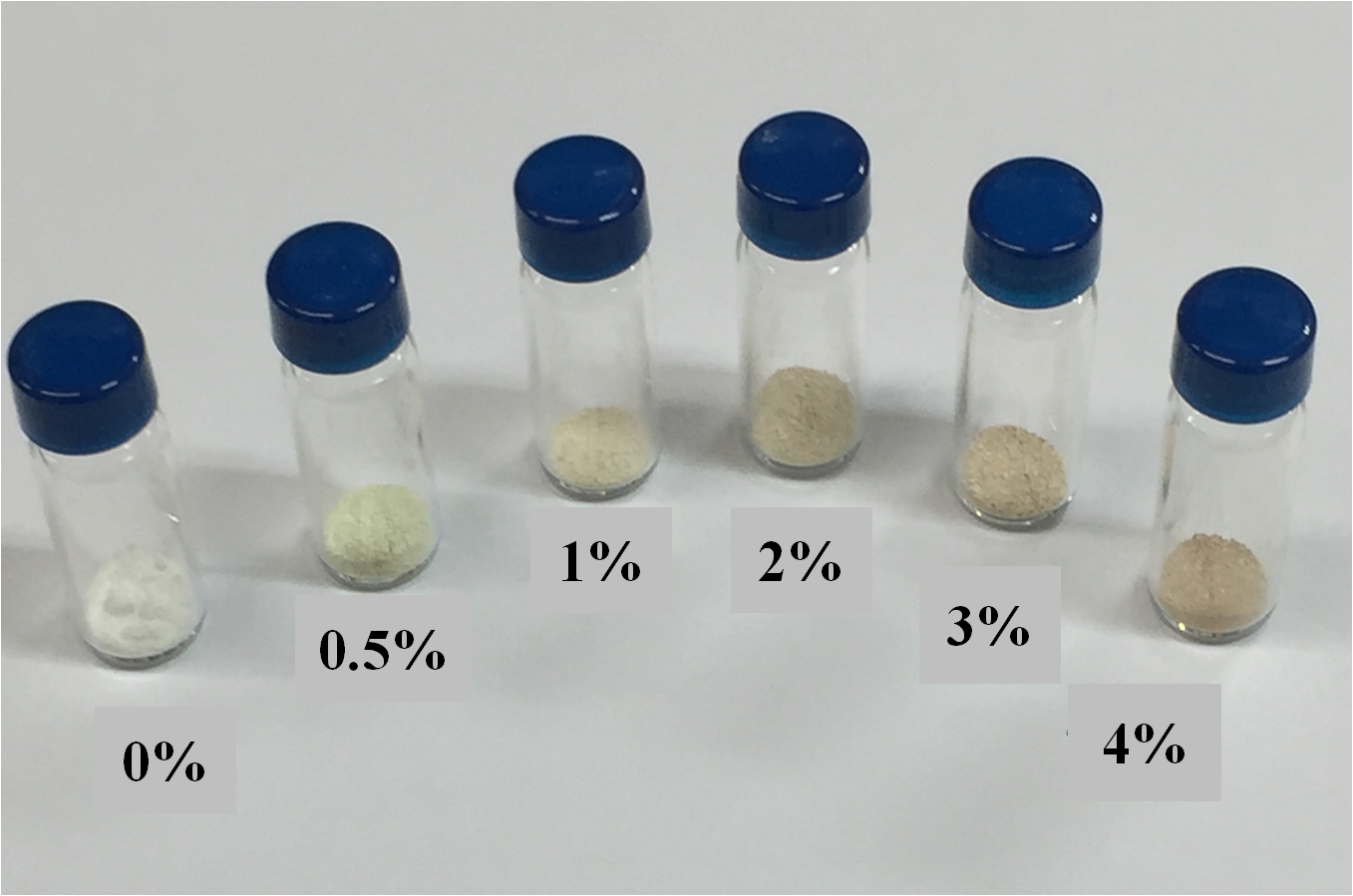


Figure. S2. Picture of as prepared BC composites.

Figure. S3. XRD pattern of as prepared N-CDs.

Figure. S4. Zeta-potential of N-CDs, BC-0 and BC-1.

Figure. S5. N_2_ adsorption/desorption isotherms of N-CDs.

Figure. S6. N_2_ adsorption/desorption isotherms of N-CDs.
